# Iterative methods of strong convergence theorems for the split feasibility problem in Hilbert spaces

**DOI:** 10.1186/s13660-016-1228-4

**Published:** 2016-11-17

**Authors:** Yuchao Tang, Liwei Liu

**Affiliations:** Department of Mathematics, Nanchang University, Nanchang, 330031 China

**Keywords:** 90C25, 90C30, 47J25, split feasibility problem, strong convergence, the best approximation

## Abstract

In this paper, we propose several new iterative algorithms to solve the split feasibility problem in the Hilbert spaces. By virtue of new analytical techniques, we prove that the iterative sequence generated by these iterative procedures converges to the solution of the split feasibility problem which is the best close to a given point. In particular, the minimum-norm solution can be found via our iteration method.

## Introduction

The split feasibility problem (SFP) was first introduced by Censor and Elfving [1] in the finite-dimensional space, which could be formulated as follows:

1.1 $$ \mbox{Finding } x\in C,\quad \mbox{such that } Ax\in Q, $$

where *C* and *Q* are nonempty closed convex subset of Hilbert space $H_{1}$ and $H_{2}$, respectively. $A:H_{1} \rightarrow H_{2}$ is a bounded linear operator. The split feasibility problem (1.1) has received much attention not only because it can be used to model the problem in signal and image processing, but also it is strongly related to some general problems, such as the convex feasibility problem [2], the multiple-set split feasibility problem [3], the split equality problem [4], the split common fixed point problem [5], etc.

Throughout the paper, we always assume that the SFP (1.1) is consistent, and Ω denotes the solution set of SFP (1.1), *i.e.*,

$$\Omega= \{ x\in C: Ax\in Q \} = C \cap A^{-1}Q. $$

To solve the SFP (1.1), Byrne [6, 7] first introduced the so-called CQ algorithm as follows:

1.2 $$ \textstyle\begin{cases} \mbox{For any } x_{0} \in H_{1}, \\ x_{n+1} = P_{C} \bigl(I - \gamma A^{*}(I-P_{Q})A \bigr)x_{n},\quad n\geq0, \end{cases} $$

where $0< \gamma< 2/\rho(A^{*}A)$, and where $P_{C}$ denotes the projection onto *C*, and $\rho(A^{*}A)$ is the spectral radius of the self-adjoint operator $A^{*}A$. In the CQ algorithm (1.2), the orthogonal projections $P_{C}$ and $P_{Q}$ have to be calculated, however, it may be impossible or one may need much time to compute in some cases. Yang [8] proposed a relaxed CQ algorithm for solving the SFP (1.1) in which the orthogonal projections $P_{C}$ and $P_{Q}$ are replaced by $P_{C_{n}}$ and $P_{Q_{n}}$, respectively, that is, the orthogonal projections onto two half spaces $C_{n}$ and $Q_{n}$. The relaxed CQ algorithm via the formula

1.3 $$ \textstyle\begin{cases} \mbox{For any } x_{0} \in H_{1}, \\ x_{n+1} = P_{C_{n}} \bigl(I - \gamma A^{*}(I-P_{Q_{n}})A \bigr)x_{n},\quad n\geq 0, \end{cases} $$

where $0< \gamma< 2/\rho(A^{*}A)$. The relaxed CQ algorithm is the use of halfspace-relaxation projection techniques due to Fukushina [9]. The half spaces $C_{n}$ and $Q_{n}$ contain the closed convex set *C* and *Q*, respectively. There is an explicit form of computing the orthogonal projection onto the half spaces $C_{n}$ and $Q_{n}$. Both the CQ algorithm and the relaxed CQ algorithm used a fixed step size and need to know the largest eigenvalues of the operator $A^{*}A$. Qu and Xiu [10] developed a modification of the relaxed CQ algorithm by adopting the Armijo-like search method. There is no need to know the largest eigenvalue of the operator $A^{*}A$ in advance, and a sufficient decrease of the objective function is done at each iteration. See for instance [11–16] and the references therein. Xu [17] presented the following averaged CQ algorithm and recall its convergence can be deduced from the averaged nonexpansiveness in [18]:

1.4 $$ \textstyle\begin{cases} \mbox{For any } x_{0} \in H_{1}, \\ x_{n+1} = (1-\alpha_{n})x _{n} + \alpha_{n} P_{C} \bigl(I - \gamma A^{*}(I-P_{Q})A \bigr)x_{n}, \quad n\geq0, \end{cases} $$

where $\{\alpha_{n}\}$ is a sequence in $[0, 4/(2+\gamma L)]$ and satisfies the condition

$$\sum_{n=0}^{\infty}\alpha_{n} \biggl( \frac{4}{2+\gamma L} -\alpha _{n} \biggr) = +\infty,\quad L = \rho \bigl(A^{*}A\bigr). $$

Since the CQ algorithm (1.2), the relaxed CQ algorithm (1.3) and the averaged CQ algorithm (1.4) have only weak convergence in the infinite-dimensional space (except in the finite-dimensional space). In order to obtain strong convergence, Xu [17] proposed the following algorithm which was inspired by the Halpern iteration method. Let $u\in H_{1}$, for any $x_{0}\in H_{1}$, the sequence $\{x_{n}\}$ is given by

1.5 $$ x_{n+1} = \alpha_{n} u + (1- \alpha_{n})P_{C}\bigl(x_{n} - \gamma A^{*}(I-P _{Q})Ax_{n}\bigr),\quad n\geq0, $$

where $0< \gamma< 2/\rho(A^{*}A)$, and the parameter $\{\alpha_{n} \}\subset(0,1)$ satisfy the conditions:

1. $\lim_{n\rightarrow\infty}\alpha_{n} = 0$, $\sum_{n=0}^{\infty }\alpha_{n} = + \infty$;
2. either $\sum_{n=0}^{\infty} \vert \alpha_{n+1}-\alpha _{n}\vert < + \infty $, or $\lim_{n\rightarrow\infty}(\alpha_{n} / \alpha_{n+1})=1$.

He proved that the sequence $\{x_{n}\}$ converges strongly to the projection of *u* onto the solution set of the SFP (1.1). In particular, if $u=0$, the iterative sequence (1.5) converges strongly to the minimum-norm solution of the SFP. Recently, Lopez et al. [19] proposed an iterative algorithm which can self-adaptive update the step size as follows:

1.6 $$ x_{n+1} = \alpha_{n} u + (1- \alpha_{n})P_{C}\bigl(x_{n} - \gamma_{n} \nabla f(x_{n})\bigr),\quad n\geq0, $$

where $\gamma_{n} = \frac{\rho_{n} f(x_{n})}{\Vert \nabla f(x_{n})\Vert ^{2}}$, $f(x)$ and $\nabla f(x)$ are defined by (2.1) and (2.2), respectively. The parameters $\{\alpha_{n}\}\subset (0,1)$ and $\{\rho_{n} \}$ satisfy the conditions

(i) $\lim_{n\rightarrow\infty}\alpha_{n} = 0$, $\sum_{n=0}^{\infty }\alpha_{n} = +\infty$;
(ii) $0< \rho_{n} < 4$, $\inf_{n\geq0}\rho_{n} (4-\rho_{n})\geq0$.

They proved that the sequence $\{x_{n}\}$ (1.6) converges strongly to $P_{\Omega}$u. Yao *et al.* [20] developed a self-adaptive iteration method to approximate the common solution of the split feasibility problem and variational inequality problem. Based on the Tikhonov regularization method, Xu [18] proved the following iterative sequence converges strongly to the minimum-norm solution of the SFP (1.1):

1.7 $$ x_{n+1} = P_{C}\bigl((1-\alpha_{n} \gamma_{n})x_{n} - \gamma_{n} A^{*}(I-P _{Q})Ax_{n}\bigr), \quad n\geq0, $$

where $\{\alpha_{n}\}$ and $\{\gamma_{n}\}$ satisfy the conditions:

(i) $0<\gamma_{n} < \frac{\alpha_{n}}{L+\alpha_{n}}$, $L = \rho(A ^{*}A)$;
(ii) $\alpha_{n} \rightarrow0$ and $\gamma_{n} \rightarrow0$ as $n\rightarrow\infty$;
(iii) $\sum_{n=0}^{\infty}\alpha_{n} \gamma_{n} = \infty$;
(iv) $(\vert \gamma_{n+1}-\gamma_{n}\vert +\gamma_{n} \vert \alpha_{n+1}-\alpha_{n}\vert )/( \alpha_{n+1}\gamma_{n+1})^{2} \rightarrow\infty$ as $n\rightarrow \infty$.

Yao *et al.* [21] proved the strong convergence of (1.7) under some different control conditions on the iterative parameters. Wang and Xu [22] proposed a modified CQ algorithm with the sequence $\{x_{n}\}$ is defined by the following:

1.8 $$ x_{n+1} = P_{C} \bigl((1-\alpha_{n}) \bigl(I - \gamma A^{*}(I - P_{Q})A\bigr)x_{n} \bigr),\quad n\geq0, $$

where $\{\alpha_{n}\}\subset(0,1)$ such that (C1)-(C2). They introduced an approximation curve for the SFP (1.1) and obtained the minimum-norm solution of the SFP as the strong limit of the approximation curve. Dang and Gao [23] introduced an iterative algorithm which combined the Krasnoselskij-Mann iterative algorithm and (1.8). The sequence $\{x_{n}\}$ is presented as follows:

1.9 $$ x_{n+1} = (1-\beta_{n})x_{n} + \beta_{n} P_{C} \bigl[ (1-\alpha_{n}) \bigl(x_{n} - \gamma A^{*} (I- P_{Q})Ax_{n} \bigr) \bigr] ,\quad n\geq0, $$

where $\gamma\in(0, 2/\rho(A^{*}A))$ and $\{\alpha_{n}\}$, $\{\beta_{n}\}$ are the sequences in $(0,1)$ such that

(i) $\lim_{n\rightarrow\infty}\alpha_{n} =0$ and $\sum_{n=0}^{ \infty}\alpha_{n} = + \infty$;
(ii) $\lim_{n\rightarrow\infty} \vert \alpha_{n} - \alpha _{n+1}\vert = 0$;
(iii) $0 < \liminf_{n\rightarrow\infty}\beta_{n} \leq \limsup_{n\rightarrow\infty}\beta_{n} < 1$.

They proved the sequence $\{x_{n}\}$ strongly converges to the solution of SFP (1.1) with no need for constructing an approximation curve in advance. It is observed that the condition (ii) is redundant as it can be deduced by condition (i). To study the variable step size of *γ*, Wand and Xu [24] proposed the following two iterative algorithms to solve the SFP (1.1). Let $u\in H$, for any $x_{0}\in H_{1}$, define

1.10 $$ x_{n+1} = \alpha_{n} u + (1- \alpha_{n}) P_{C}\bigl(x_{n} - \lambda_{n} A ^{*}(I-P_{Q})Ax_{n} \bigr), \quad n\geq0, $$

and

1.11 $$ x_{n+1} = P_{C}\bigl(\alpha_{n} u + (1-\alpha_{n}) \bigl( x_{n} - \lambda_{n} A ^{*}(I - P_{Q})Ax_{n} \bigr)\bigr),\quad n\geq0, $$

where the sequences $\{\lambda_{n}\}$ and $\{\alpha_{n}\}$ satisfy the following conditions:

(i) $0< a \leq\gamma_{n} \leq b < \frac{2}{L}$, $L=\rho(A^{*}A)$;
(ii) $\sum_{n=0}^{\infty} \vert \lambda_{n+1}-\lambda_{n}\vert < + \infty$;
(iii) $\lim_{n\rightarrow\infty}\alpha_{n} =0$, $\sum_{n=0}^{\infty }\alpha_{n} = +\infty$;
(iv) either $\sum_{n=0}^{\infty} \vert \alpha_{n+1}-\alpha _{n}\vert < +\infty $ or $\lim_{n\rightarrow\infty} \vert \alpha_{n+1}-\alpha_{n} \vert / \alpha _{n} =0$.

They proved the sequence generated by (1.10) and (1.11) converge strongly to $P_{\Omega}u$. Further, in [25], Yao *et al.* proposed an iterative algorithm to solve the common solution of the split feasibility problem and fixed point problem. They proved the strong convergence of the proposed iterative algorithm. See also [26–29].

Motivated and inspired by the above work, we will continue to study the strong convergence method to solve the SFP (1.1). We propose two iteration methods to do such a job. Let $u\in H_{1}$, for any $x_{0}\in H_{1}$, the first iterative sequence $\{x_{n}\}$ is defined by the following procedure:

1.12 $$ x_{n+1} = (1-\alpha_{n})x_{n} + \alpha_{n} P_{C}\bigl(t_{n} u + (1-t_{n})U _{n} x_{n}\bigr),\quad n\geq0, $$

and the second iterative sequence $\{x_{n}\}$ is given as follows:

1.13 $$ x_{n+1} = (1-\alpha_{n})x_{n} + \alpha_{n} \bigl(t_{n} u + (1-t_{n})P_{C}U _{n} x_{n}\bigr),\quad n\geq0, $$

where $U_{n} = I - \gamma_{n} A^{*}(I- P_{Q})A$, $\{\alpha_{n}\}, \{t _{n}\}\subset(0,1)$, and $\{\gamma_{n}\}$ satisfy the condition

1.14 $$ 0 < \liminf_{n\rightarrow\infty}\gamma_{n} \leq \limsup_{n\rightarrow \infty}\gamma_{n} < 2/L,\quad L = \rho \bigl(A^{*}A\bigr). $$

Under the assumptions on the parameters $\{\alpha_{n}\}$ and $\{t_{n}\}$, we prove that the iteration sequence $\{x_{n}\}$ generated by (1.12) and (1.13) converges strongly to the projection of *u* onto the solution set of the SFP (1.1).

## Preliminaries

In this section, we collect some important definitions and some useful lemmas which will be used in the following section. Let *H* be a real Hilbert space with inner product $\langle\cdot, \cdot\rangle$ and norm $\Vert \cdot \Vert $, respectively. We introduce the following notations.

(i) The set of all fixed point of *T* is denoted by $\operatorname{Fix}(T)$.
(ii) The symbol ⇀ for weak convergence and → for strong convergence, respectively.

The following definitions are well known.

### Definition 2.1

Let *C* be a nonempty closed convex subset of *H*. $T:C\rightarrow C$ is called

(i) a nonexpansive mapping, if $\Vert Tx-Ty\Vert \leq \Vert x-y\Vert $, for all $x,y\in C$,
(ii) a firmly nonexpansive mapping, if $\Vert Tx-Ty\Vert ^{2} \leq \langle x-y, Tx-Ty \rangle$, for all $x,y\in C$,
(iii) an *α*-averaged nonexpansive mapping, if there exists a nonexpansive mapping *S*, such that $T = (1-\alpha)I + \alpha S$, where $\alpha\in(0,1)$ and *I* is the identity mapping.

Recall that the orthogonal projection $P_{C}x$ from *H* onto a nonempty closed convex subset $C\subset H$ is defined by the following:

$$P_{C}x = \arg\min_{y\in C} \Vert x-y\Vert . $$

The orthogonal projection has the following well-known properties. For a given $x\in H$,

(i) $\langle x - P_{C}x, z- P_{C}x \rangle\leq0$, for all $z\in C$;
(ii) $\Vert P_{C}x - P_{C}y\Vert ^{2} \leq\langle P_{C}x - P_{C}y, x-y \rangle$, for all $x, y \in H$.

### Remark 2.1

It is easy to see that the projection operator is a firmly nonexpansive mapping. The relation between projection operator, firm nonexpansiveness, averaged nonexpansiveness, and nonexpansiveness can be presented as follows.

$$\begin{aligned} \mbox{Projecton operator} \Rightarrow&\mbox{Firmly nonexpansive} \Rightarrow \mbox{Averaged nonexpansive} \\ \Rightarrow& \mbox{Nonexpansive}. \end{aligned}$$

The CQ algorithm (1.2) can be viewed from two different but equivalent ways: optimization and fixed point. See, for example [18]. To solve the SFP (1.1) from the point of view optimization. Define the proximity function

2.1 $$ f(x) = \frac{1}{2}\Vert Ax-P_{Q}Ax \Vert ^{2}. $$

Then the gradient of $f(x)$ is

2.2 $$ \nabla f(x) = A^{*}(Ax - P_{Q}Ax). $$

In addition, ∇*f* is Lipschitz continuous, with Lipschitz constant $L = \rho(A^{*}A)$. The fixed point method approach to solve the SFP (1.1) is based on the fact that the SFP (1.1) can be formulated as a fixed point equation.

### Lemma 2.1

[18, 23]

*Suppose the* $\Omega\neq\emptyset$, *let* $U = I - \gamma A^{*}(I-P _{Q})A$, $0< \gamma< 2/L$, $L= \rho(A^{*}A)$, *and* $T := P_{C}U$. *Then*

(i) *U* *is an* $\frac{\gamma L}{2}$-*averaged nonexpansive mapping*.
(ii) $\operatorname{Fix}(T) = \operatorname{Fix}(P_{C}) \cap \operatorname{Fix}(U) = \Omega$.

### Remark 2.2

Define $U_{n} = I - \gamma_{n} A^{*}(I-P_{Q})A$, $T_{n} = P_{C}U_{n}$, where the parameter $\{\gamma_{n}\}$ satisfies the condition (1.14), then the mappings $U_{n} $ is also an $\frac{\gamma _{n} L}{2}$-averaged nonexpansive mapping, and $\operatorname{Fix}(T_{n}) = \operatorname{Fix}(P _{C})\cap \operatorname{Fix}(U_{n}) = \Omega$.

The nonexpansive mapping has the following important demiclosedness property. Other important properties of nonexpansive mapping can be found in [30–32].

### Lemma 2.2

*Let* $T:C\rightarrow C$ *is a nonexpansive mapping with* $\operatorname{Fix}(T)\neq \emptyset$. *If* $x_{n} \rightharpoonup x$ *and* $(I-T)x_{n} \rightarrow 0$, *then* $x=Tx$.

We need the following technical lemmas to facilitate our proof. The lemma below was used by many authors as the key tool in proving convergence theorems. See also [33, 34].

### Lemma 2.3

[35]

*Let* $\{x_{n}\}$ *and* $\{y_{n}\}$ *be bounded sequences in a Banach space* *E* *and let* $\{\beta_{n}\}$ *be a sequence in* $[0,1]$ *with* $0< \liminf_{n\rightarrow\infty}\beta_{n} \leq \limsup_{n\rightarrow\infty}\beta_{n} <1$. *Suppose* $x_{n+1} = \beta _{n} y_{n} + (1-\beta_{n})x_{n}$ *for all* $n\geq0$ *and*

$$\limsup_{n\rightarrow\infty} \bigl( \Vert y_{n+1} - y_{n} \Vert - \Vert x_{n+1} -x_{n}\Vert \bigr) \leq0. $$

*Then* $\lim_{n\rightarrow\infty} \Vert y_{n} -x_{n}\Vert =0$.

We shall use the following recurrent inequality to obtain our strong convergence theorems.

### Lemma 2.4

[36]

*Let* $\{a_{n}\}$ *be a sequence of non*-*negative real sequences satisfying the following inequality*:

$$a_{n+1} \leq(1-\gamma_{n})a_{n} + \gamma_{n} \delta_{n},\quad n\geq0, $$

*where* $\{\gamma_{n}\}$ *is a sequence in* $(0,1)$ *and* $\{\delta_{n}\}$ *is a sequence such that*

1. $\sum_{n=0}^{\infty}\gamma_{n} = +\infty$;
2. *either* $\limsup_{n\rightarrow\infty}\delta_{n} \leq0$ *or* $\sum_{n=0}^{\infty} \vert \gamma_{n} \delta_{n}\vert < +\infty$.

*Then* $\lim_{n\rightarrow\infty}a_{n} = 0$.

The following proposition presents some important equality and inequality properties that hold in any Hilbert space. We refer to [32] for other properties in a Hilbert space.

### Proposition 2.1

*Let* *H* *be a Hilbert space with inner product* $\langle\cdot, \cdot \rangle$ *and norm* $\Vert \cdot \Vert $, *respectively*. *Then*

(i) $\Vert x+y\Vert ^{2} = \Vert x\Vert ^{2} + 2\langle x,y\rangle+ \Vert y \Vert ^{2}$,
(ii) $\Vert x+y\Vert ^{2} \leq \Vert x\Vert ^{2} + 2 \langle x+y,y \rangle$,
(iii) $\bigl \Vert \alpha x + (1-\alpha)y \bigr \Vert ^{2} = \alpha \Vert x\Vert ^{2} + (1- \alpha)\Vert y\Vert ^{2} - \alpha(1-\alpha)\Vert x-y\Vert ^{2}$,

$\forall x,y\in H$ *and* $\forall\alpha\in[0,1]$.

## Main results

In this section, we state and prove our main results. First, we prove the strong convergence of the iterative sequence (1.12).

### Theorem 1

*Assume that the SFP* (1.1) *is consistent* (*i*.*e*., *the solution set* Ω *is nonempty*). *Let the sequence* $\{x_{n}\}_{n=0} ^{\infty}$ *be defined by* (1.12), *where the parameters* $\{\alpha_{n}\}$ *and* $\{t_{n}\} \subset(0,1)$ *satisfy the following conditions*:

(i) $0< \liminf_{n\rightarrow\infty}\alpha_{n} \leq \limsup_{n\rightarrow\infty}\alpha_{n} <1$;
(ii) $\lim_{n\rightarrow\infty}t_{n} =0$, $\sum_{n=0}^{\infty}t _{n} = +\infty$.

*In addition the parameter* $\{\gamma_{n}\}$ *satisfies* $\lim_{n\rightarrow\infty} \vert \gamma_{n+1}-\gamma_{n}\vert =0$. *Then the sequence* $\{x_{n}\}$ *converges strongly to the point of* *u* *onto the projection of* Ω, *i*.*e*., $x_{n} \rightarrow P_{\Omega }u$.

### Proof

Let $z_{n} = P_{C}(t_{n} u + (1-t_{n})U_{n} x_{n})$, then the iterative sequence (1.12) can be rewritten as

3.1 $$ x_{n+1} = (1-\alpha_{n})x_{n} + \alpha_{n} z_{n}. $$

Let $p\in\Omega$. By Lemma 2.1, we know that $p\in C$ and $p\in \operatorname{Fix}(U_{n})$. Then we have

3.2 $$\begin{aligned} \Vert x_{n+1} - p \Vert =& \bigl\Vert (1- \alpha_{n}) (x_{n} - p) + \alpha_{n} (z_{n} -p) \bigr\Vert \\ \leq&(1-\alpha_{n})\Vert x_{n} - p \Vert + \alpha_{n} \bigl\Vert t_{n} u + (1-t_{n})U_{n}x_{n} - p \bigr\Vert \\ = &(1-\alpha_{n})\Vert x_{n} - p\Vert + \alpha_{n} \bigl\Vert t_{n} (u-p) + (1-t_{n}) (U_{n}x_{n} - p) \bigr\Vert \\ \leq&(1-\alpha_{n})\Vert x_{n} - p \Vert + \alpha_{n} t_{n} \Vert u-p\Vert + \alpha_{n} (1-t_{n})\Vert x_{n} -p \Vert \\ =& (1-\alpha_{n} t_{n})\Vert x_{n} -p\Vert + \alpha_{n} t_{n} \Vert u-p\Vert . \end{aligned}$$

By induction, it follows from (3.2) that

$$\Vert x_{n+1} -p \Vert \leq\max\bigl\{ \Vert x_{0} -p \Vert , \Vert u-p\Vert \bigr\} , $$

which means that the sequence $\{x_{n}\}$ is bounded.

Next, we prove that $\Vert x_{n+1}-x_{n}\Vert \rightarrow0$ as $n\rightarrow \infty$. Notice that $z_{n} = P_{C}(t_{n} u + (1-t_{n})U_{n} x_{n})$ and $p\in \operatorname{Fix}(U_{n})$, we have

3.3 $$\begin{aligned} \Vert z_{n} - p \Vert =& \bigl\Vert P_{C} \bigl(t_{n} u + (1-t_{n})U_{n} x_{n} \bigr) - p \bigr\Vert \\ \leq&\bigl\Vert t_{n} u + (1-t_{n})U_{n} x_{n} - p \bigr\Vert \\ \leq &t_{n} \Vert u-p\Vert + (1-t_{n})\Vert x_{n} -p\Vert \\ \leq&\max\bigl\{ \Vert x_{n} -p\Vert , \Vert u-p\Vert \bigr\} . \end{aligned}$$

Since the sequence $\{x_{n}\}$ is bounded, the sequence $\{z_{n}\}$ is also bounded. Again, from the Lipschitz continuous of $A^{*}(I-P_{Q})Ax _{n}$ and $U_{n} x_{n}$, there exists a constant $M>0$ such that

$$M > \max\Bigl\{ \sup_{n\geq0}\bigl\Vert A^{*}(I-P_{Q})Ax_{n} \bigr\Vert , \sup_{n\geq 0}\Vert x_{n}\Vert , \sup _{n\geq0}\Vert U_{n} x_{n}\Vert \Bigr\} . $$

From the nonexpansivity of the projection operator $P_{C}$, we have

3.4 $$\begin{aligned} \Vert z_{n+1} - z_{n} \Vert =& \bigl\Vert P_{C}\bigl(t_{n+1}u +(1-t_{n+1})U_{n+1}x_{n+1} \bigr) - P_{C}\bigl(t_{n} u + (1-t_{n})U_{n} x_{n}\bigr) \bigr\Vert \\ \leq&\bigl\Vert t_{n+1}u + (1-t_{n+1})U_{n+1}x_{n+1} - \bigl(t_{n} u + (1-t_{n})U_{n}x_{n} \bigr) \bigr\Vert \\ \leq&\vert t_{n+1}-t_{n} \vert \Vert u\Vert + \bigl\Vert (1-t_{n+1})U_{n+1}x_{n+1} -(1-t_{n})U_{n} x_{n} \bigr\Vert \\ \leq&\vert t_{n+1}-t_{n} \vert \Vert u\Vert + \bigl\Vert (1-t_{n+1})U_{n+1}x_{n+1} -(1-t_{n+1})U_{n+1}x_{n} \bigr\Vert \\ &{}+ \bigl\Vert (1-t_{n+1})U_{n+1}x_{n} - (1-t_{n})U_{n+1}x_{n} \bigr\Vert \\ &{} + \bigl\Vert (1-t_{n})U_{n+1}x_{n} - (1-t_{n})U_{n} x_{n} \bigr\Vert \\ \leq&\vert t_{n+1} - t_{n} \vert \Vert u\Vert + (1-t_{n+1})\Vert x_{n+1}-x_{n}\Vert \\ &{} + \vert t_{n+1}-t_{n}\vert M + (1-t_{n}) \Vert U_{n+1}x_{n} - U_{n} x_{n} \Vert \\ \leq&\vert t_{n+1} - t_{n} \vert \Vert u\Vert + (1-t_{n+1})\Vert x_{n+1}-x_{n}\Vert \\ &{} + \vert t_{n+1}-t_{n}\vert M + (1-t_{n}) \vert \gamma _{n+1}-\gamma_{n}\vert M, \end{aligned}$$

which implies that

$$\begin{aligned}& \Vert z_{n+1} -z_{n} \Vert - \Vert x_{n+1}-x_{n}\Vert \\& \quad \leq \vert t_{n+1} - t_{n} \vert \Vert u\Vert + \vert t_{n+1} - t_{n} \vert M + (1-t_{n}) \vert \gamma_{n+1} - \gamma_{n} \vert M. \end{aligned}$$

By the assumptions of (i) and (ii), we have

$$\limsup_{n\rightarrow\infty} \bigl( \Vert z_{n+1} - z_{n} \Vert - \Vert x_{n+1}-x_{n}\Vert \bigr) \leq0. $$

One concludes from Lemma 2.3 that

$$\lim_{n\rightarrow\infty} \Vert x_{n} - z_{n} \Vert = 0. $$

Therefore,

$$\lim_{n\rightarrow\infty} \Vert x_{n+1}-x_{n}\Vert = \lim_{n\rightarrow \infty} \alpha_{n} \Vert x_{n} - z_{n}\Vert = 0. $$

Next, we make the following estimation:

3.5 $$\begin{aligned}& \bigl\Vert x_{n} - P_{C}(U_{n} x_{n}) \bigr\Vert \\& \quad \leq \Vert x_{n} - x_{n+1} \Vert + \bigl\Vert x_{n+1} - P_{C}(U_{n} x_{n}) \bigr\Vert \\& \quad \leq \Vert x_{n} - x_{n+1} \Vert + \bigl\Vert (1- \alpha_{n} )x_{n} + \alpha_{n}P_{C} \bigl(t_{n} u + (1-t_{n})U_{n} x_{n} \bigr) - P_{C}(U_{n} x_{n}) \bigr\Vert \\& \quad \leq \Vert x_{n} - x_{n+1} \Vert + (1- \alpha_{n}) \bigl\Vert x_{n} - P_{C}(U_{n} x_{n})\bigr\Vert \\& \qquad {} + \alpha_{n} \bigl\Vert t_{n} u + (1-t_{n} U_{n} x_{n}) - U_{n} x_{n} \bigr\Vert \\& \quad \leq \Vert x_{n} - x_{n+1} \Vert + (1- \alpha_{n}) \bigl\Vert x_{n} - P_{C}(U_{n} x_{n})\bigr\Vert + \alpha_{n} t_{n} \Vert u - U_{n} x_{n}\Vert . \end{aligned}$$

It turns out that

3.6 $$ \bigl\Vert x_{n} - P_{C}(U_{n})x_{n} \bigr\Vert \leq\frac{1}{\alpha_{n}} \Vert x_{n} - x_{n+1} \Vert + t_{n} \Vert u - U_{n} x_{n}\Vert . $$

We show that $\limsup_{n\rightarrow\infty}\langle U_{n} x_{n} - q, u -q \rangle\leq0$, where $q = P_{\Omega}u$. It is easy to see that

3.7 $$\begin{aligned} \langle U_{n} x_{n} - q, u -q \rangle =& \langle U_{n} x_{n} - x_{n}, u - q \rangle+ \langle x_{n} -q, u - q \rangle \\ \leq&\Vert U_{n} x_{n} - x_{n}\Vert \Vert u-q\Vert + \langle x_{n} -q, u -q \rangle. \end{aligned}$$

For any $p\in\Omega$, we have

3.8 $$\begin{aligned} \Vert x_{n+1} - p \Vert ^{2} =& \bigl\Vert (1-\alpha_{n})x_{n} + \alpha_{n} P_{C}\bigl(t_{n} u+ (1-t_{n})U_{n} x_{n}\bigr) - p \bigr\Vert ^{2} \\ \leq&(1-\alpha_{n})\Vert x_{n} - p\Vert ^{2} + \alpha_{n} \bigl\Vert P_{C}\bigl(t_{n} u+(1-t_{n})U_{n} x_{n}\bigr) - p \bigr\Vert ^{2} \\ \leq&(1-\alpha_{n})\Vert x_{n} - p\Vert ^{2} + \alpha_{n} \bigl( t_{n} \Vert u-p\Vert ^{2} + (1-t_{n})\Vert U_{n} x_{n} - p \Vert ^{2} \bigr). \end{aligned}$$

By Lemma 2.1, we know that $U_{n}$ is averaged nonexpansive, that is, $U_{n} = (1-\beta_{n})I + \beta_{n} V_{n}$, where $\beta_{n} = \frac{\gamma_{n} L}{2}$. Then

3.9 $$\begin{aligned} \Vert U_{n} x_{n} -p \Vert ^{2} = &\bigl\Vert (1-\beta_{n}) (x_{n} -p) + \beta_{n} (V_{n} x_{n}-p) \bigr\Vert ^{2} \\ =& (1-\beta_{n})\Vert x_{n} -p\Vert ^{2} + \beta_{n} \Vert V_{n} x_{n} - p \Vert ^{2} - \beta_{n} (1-\beta_{n})\Vert x_{n} - V_{n} x_{n}\Vert ^{2} \\ \leq&\Vert x_{n} -p\Vert ^{2} - \beta_{n} (1-\beta_{n})\Vert x_{n} - V_{n} x_{n}\Vert ^{2}. \end{aligned}$$

Substituting (3.9) into (3.8), we obtain

3.10 $$\begin{aligned} \Vert x_{n+1}-p\Vert ^{2} \leq&(1-\alpha_{n}) \Vert x_{n} -p\Vert ^{2} + \alpha_{n} \Vert x_{n}-p\Vert ^{2} \\ &{} - \alpha_{n} \beta_{n} (1-\beta_{n})\Vert x_{n} - V_{n} x_{n}\Vert ^{2} + \alpha_{n} t_{n} \Vert u-p\Vert ^{2} \\ =& \Vert x_{n} -p\Vert ^{2} - \alpha_{n} \beta_{n} (1-\beta_{n}) \Vert x_{n} - V_{n}x_{n}\Vert ^{2} + \alpha_{n} t_{n} \Vert u-p\Vert ^{2}. \end{aligned}$$

Therefore,

3.11 $$\begin{aligned} \alpha_{n} \beta_{n} (1-\beta_{n}) \Vert x_{n} - V_{n} x_{n}\Vert ^{2} \leq& \Vert x_{n}-p\Vert ^{2} - \Vert x_{n+1}-p \Vert ^{2} + \alpha_{n} t_{n} \Vert u-p\Vert ^{2} \\ \leq&\bigl( \Vert x_{n} -p\Vert - \Vert x_{n+1}-p \Vert \bigr) \bigl( \Vert x_{n} -p\Vert + \Vert x_{n+1}-p\Vert \bigr) \\ &{}+ \alpha_{n} t_{n} \Vert u-p\Vert ^{2} \\ \leq&\Vert x_{n} - x_{n+1}\Vert 2\bigl(M + \Vert p \Vert \bigr) + \alpha_{n} t_{n} \Vert u-p\Vert ^{2}. \end{aligned}$$

Then

$$\beta_{n} (1-\beta_{n})\Vert x_{n} - V_{n} x_{n}\Vert ^{2} \leq \frac{ \Vert x_{n}-x_{n+1}\Vert }{\alpha_{n}}2 \bigl(M + \Vert p\Vert \bigr) + t_{n} \Vert u-p \Vert ^{2} $$

and

3.12 $$ \lim_{n\rightarrow\infty} \Vert U_{n} x_{n} - x_{n}\Vert = \lim_{n\rightarrow \infty} \beta_{n} \Vert x_{n} - V_{n} x_{n} \Vert =0. $$

We can choose a subsequence $\{x_{n_{j}}\}$ of $\{x_{n}\}$ such that

$$\limsup_{n\rightarrow\infty} \langle x_{n} - q, u -q \rangle= \lim _{j\rightarrow\infty} \langle x_{n_{j}} - q, u -q \rangle. $$

Since $\{x_{n_{j}}\}$ is bounded, there exists a subsequence of $\{x_{n_{j}}\}$ which converges weakly to a point *x̅*. Without loss of generality, we may assume that $x_{n_{j}}\rightharpoonup \overline{x}$. Since $\{\gamma_{n}\}$ is bounded, we may assume $\gamma_{n_{j}}\rightarrow\gamma$. Let $U = I - \gamma A^{*}(I-P _{Q})A$, we have

3.13 $$\begin{aligned} \bigl\Vert x_{n_{j}} - P_{C}(U x_{n_{j}}) \bigr\Vert & \leq\bigl\Vert x_{n_{j}} -P_{C}(U_{n_{j}}x_{n_{j}}) \bigr\Vert + \bigl\Vert P_{C}(U_{n_{j}}x_{n_{j}}) - P_{C}(Ux_{n_{j}}) \bigr\Vert \\ & \leq\bigl\Vert x_{n_{j}} - P_{C}(U_{n_{j}}x_{n_{j}}) \bigr\Vert + \Vert U_{n_{j}}x_{n_{j}} -U x_{n_{j}} \Vert \\ & \leq\bigl\Vert x_{n_{j}} - P_{C}(U_{n_{j}}x_{n_{j}}) \bigr\Vert + \vert \gamma_{n_{j}}-\gamma \vert M \\ & \rightarrow0, \quad \mbox{as } j \rightarrow\infty. \end{aligned}$$

Since $P_{C}U$ is nonexpansive, from the demiclosed Lemma 2.2, we know that $\overline{x}\in \operatorname{Fix}(P_{C}U)$, that is, $\overline{x}\in\Omega$. It follows from the properties of projection operator that

3.14 $$ \limsup_{n\rightarrow\infty} \langle x_{n} - q, u -q \rangle= \langle\overline{x} - q, u -q \rangle\leq0. $$

Taking the limsup on both sides of (3.7), and together with (3.12) and (3.14), we get

3.15 $$ \limsup_{n\rightarrow\infty}\langle U_{n} x_{n} - q, u -q \rangle \leq 0. $$

Finally, we prove that $x_{n} \rightarrow q$, where $q = P_{\Omega}u$. By (1.12) and Proposition 2.1, we have

3.16 $$\begin{aligned} \Vert x_{n+1} - q \Vert ^{2} =& \bigl\Vert (1- \alpha_{n}) (x_{n} - q) + \alpha_{n} \bigl(P_{C}\bigl(t_{n} u + (1-t_{n})U_{n} x_{n}\bigr) -q \bigr) \bigr\Vert ^{2} \\ \leq&(1-\alpha_{n})\Vert x_{n} -q\Vert ^{2} + \alpha_{n} \bigl\Vert P_{C}\bigl(t_{n} u +(1-t_{n})U_{n} x_{n}\bigr) -q \bigr\Vert ^{2} \\ \leq&(1-\alpha_{n})\Vert x_{n} -q\Vert ^{2} + \alpha_{n} \bigl\Vert t_{n} (u-q) +(1-t_{n}) (U_{n} x_{n} -q) \bigr\Vert ^{2} \\ =& (1-\alpha_{n})\Vert x_{n} -q\Vert ^{2} + 2 \alpha_{n} t_{n} (1-t_{n}) \langle u-q, U_{n} x_{n} -q \rangle \\ &{} + \alpha_{n} t_{n}^{2} \Vert u-q \Vert ^{2} + \alpha_{n} (1-t_{n})^{2} \Vert U_{n} x_{n}-q \Vert ^{2} \\ \leq&(1-\alpha_{n} t_{n})\Vert x_{n} -q \Vert ^{2} + 2 \alpha _{n} t_{n} (1-t _{n})\langle u - q, U_{n} x_{n} -q \rangle \\ &{} + \alpha_{n} t_{n}^{2}\Vert u-q \Vert ^{2}. \end{aligned}$$

Let $\gamma_{n} = \alpha_{n} t_{n}$ and $\delta_{n} = 2 (1-t_{n}) \langle u-q, U_{n} x_{n} -q\rangle+ t_{n} \Vert u-q\Vert ^{2}$. Notice the condition that $\lim_{n\rightarrow\infty}t_{n} =0$ and the inequality (3.15), we have $\sum_{n=0}^{\infty}\gamma_{n} = +\infty$ and $\limsup_{n\rightarrow\infty}\delta_{n} \leq0$. By Lemma 2.4, we obtain $\Vert x_{n} -q\Vert \rightarrow0$. □

We have proved the strong convergence of iterative method (1.12). Next, we are ready to prove the corresponding convergence theorem as regards the iterative algorithm of (1.13).

### Theorem 2

*Assume that the SFP* (1.1) *is consistent* (*i*.*e*., *the solution set* Ω *is nonempty*). *Let the iterative sequence* $\{x_{n}\}$ *is defined by* (1.13), *where the iterative parameters* $\{\alpha_{n}\}$, $\{t_{n}\}$ *and* $\{\gamma_{n}\}$ *satisfy the same conditions as in Theorem* 1. *Then the sequence* $\{x_{n}\}$ *converges strongly to the point of* *u* *onto the projection of* Ω, *i*.*e*., $x_{n} \rightarrow P_{\Omega}u$.

### Proof

The principal proof of Theorem 2 is similar to Theorem 1. However, the derivation is slightly different. We give the detailed proofs as follows. Let $z_{n} = t_{n} u + (1-t_{n})P_{C}U _{n} x_{n}$, then the iterative sequence (1.13) can be formulated as follows:

3.17 $$ x_{n+1} = (1-\alpha_{n})x_{n} + \alpha_{n} z_{n}. $$

For simplicity, we separate the proof into four steps.

*Step 1.* We prove that the sequence $\{x_{n}\}$ is bounded. In fact, let $p\in\Omega$. By Lemma 2.1, we know that $p \in C$ and $p\in \operatorname{Fix}(U_{n})$. We have from (3.17)

3.18 $$\begin{aligned} \Vert x_{n+1} - p \Vert =& \bigl\Vert (1-\alpha_{n}) (x_{n} -p) + \alpha_{n} (z_{n}-p)\bigr\Vert \\ \leq&(1-\alpha_{n})\Vert x_{n} -p\Vert + \alpha_{n} \bigl\Vert t_{n} u + (1-t_{n})P_{C}U_{n}x_{n} - p\bigr\Vert \\ \leq&(1-\alpha_{n})\Vert x_{n} -p\Vert + \alpha_{n} t_{n} \Vert u-p\Vert + \alpha _{n} (1-t_{n})\Vert x_{n} -p\Vert \\ =& (1-\alpha_{n} t_{n})\Vert x_{n} -p\Vert + \alpha_{n} t_{n} \Vert u-p\Vert \\ \leq&\max\bigl\{ \Vert x_{0} -p\Vert , \Vert u-p\Vert \bigr\} . \end{aligned}$$

This means that $\{x_{n}\}$ is bounded.

*Step 2.* We show that $\Vert x_{n+1}-x_{n}\Vert \rightarrow0$ as $n\rightarrow\infty$. Since $z_{n} = t_{n} u + (1-t_{n})P_{C}U_{n} x _{n}$ and $p\in \operatorname{Fix}(U_{n})$, we have

3.19 $$\begin{aligned} \Vert z_{n} -p\Vert =& \bigl\Vert t_{n} u + (1-t_{n})P_{C}U_{n} x_{n} - p\bigr\Vert \\ \leq& t_{n} \Vert u-p\Vert + (1-t_{n})\Vert x_{n} -p\Vert \\ \leq&\max\bigl\{ \Vert x_{n} -p\Vert , \Vert u-p\Vert \bigr\} . \end{aligned}$$

Therefore, $\{z_{n}\}$ is also bounded. Let $M > 0$, such that

3.20 $$ M > \max\Bigl\{ \sup_{n\geq0}\bigl\Vert A^{*}(I - P_{Q})Ax_{n}\bigr\Vert , \sup_{n\geq0} \Vert x_{n}\Vert , \sup_{n\geq0}\Vert P_{C}U_{n} x_{n}\Vert \Bigr\} . $$

On the other hand, we have

3.21 $$\begin{aligned} \Vert z_{n+1} - z_{n} \Vert =& \bigl\Vert t_{n+1} u +(1-t_{n+1})P_{C}U_{n+1}x_{n+1}- t_{n} u - (1-t_{n})P_{C}U_{n} x_{n} \bigr\Vert \\ \leq&\vert t_{n+1}-t_{n}\vert \Vert u\Vert + \bigl\Vert (1-t_{n+1})P_{C}U_{n+1}x_{n+1} -(1-t_{n+1})P_{C}U_{n+1}x_{n} \bigr\Vert \\ &{} + \bigl\Vert (1-t_{n+1})P_{C}U_{n+1}x_{n} - (1-t_{n})P_{C}U_{n+1}x_{n} \bigr\Vert \\ &{} + \bigl\Vert (1-t_{n})P_{C}U_{n+1}x_{n} - (1-t_{n})P_{C}U_{n} x_{n} \bigr\Vert \\ \leq&\vert t_{n+1}-t_{n} \vert \Vert u\Vert + (1-t_{n+1})\Vert x_{n+1}-x_{n}\Vert \\ &{} + \vert t_{n+1}-t_{n}\vert M + (1-t_{n}) \Vert U_{n+1}x_{n} - U_{n}x_{n} \Vert \\ \leq&\vert t_{n+1}-t_{n} \vert \Vert u\Vert + (1-t_{n+1})\Vert x_{n+1}-x_{n}\Vert \\ &{} + \vert t_{n+1}-t_{n}\vert M + (1-t_{n}) \vert \gamma_{n+1} - \gamma_{n} \vert M. \end{aligned}$$

It turns out from (3.21) that

$$\begin{aligned}& \Vert z_{n+1}-z_{n}\Vert - \Vert x_{n+1}-x_{n} \Vert \\& \quad \leq \vert t_{n+1}-t_{n}\vert \Vert u\Vert + \vert t_{n+1}-t_{n}\vert M + (1-t_{n})\vert \gamma_{n+1}-\gamma _{n}\vert M. \end{aligned}$$

Taking limsup on both sides of the above inequality, we get

$$\limsup_{n\rightarrow\infty} \bigl( \Vert z_{n+1}-z_{n} \Vert - \Vert x_{n+1}-x_{n}\Vert \bigr) \leq0. $$

With the help of Lemma 2.3, we obtain $\lim_{n\rightarrow\infty} \Vert x_{n} - z_{n}\Vert =0$. Therefore,

3.22 $$ \lim_{n\rightarrow\infty} \Vert x_{n+1}-x_{n}\Vert = \lim_{n\rightarrow \infty} \alpha_{n} \Vert x_{n} -z_{n}\Vert = 0. $$

*Step 3.* We prove that $\limsup_{n\rightarrow\infty}\langle q - x_{n}, q - u \rangle\leq0$, where $q = P_{\Omega}u$. We have

3.23 $$\begin{aligned} \Vert x_{n} - P_{C}U_{n} x_{n} \Vert \leq&\Vert x_{n} - x_{n+1} \Vert + \Vert x_{n+1}-P_{C}U_{n} x_{n}\Vert \\ \leq&\Vert x_{n} - x_{n+1}\Vert + (1- \alpha_{n})\Vert x_{n} - P_{C}U_{n} x_{n}\Vert + \alpha_{n} t_{n} \bigl(M+\Vert u\Vert \bigr), \end{aligned}$$

which leads to

3.24 $$\begin{aligned} \Vert x_{n} - P_{C}U_{n} x_{n} \Vert \leq&\frac{1}{\alpha_{n}}\Vert x_{n} - x_{n+1}\Vert + t_{n} \bigl(M + \Vert u\Vert \bigr). \end{aligned}$$

We can choose a subsequence $\{x_{n_{j}}\}$ of $\{x_{n}\}$ such that

$$\limsup_{n\rightarrow\infty}\langle q -x_{n}, q -u \rangle= \lim _{j\rightarrow\infty} \langle q - x_{n_{j}}, q -u \rangle. $$

Because $\{x_{n_{j}}\}$ is bounded, there exists a subsequence of $\{x_{n_{j}}\}$ which converges weakly to a point *x̅*. Without loss of generality, we may assume that $\{x_{n_{j}}\}$ converges weakly to *x̅*. Since $\{\gamma_{n}\}$ is bounded, we may assume that $\gamma_{n_{j}}\rightarrow\gamma$. Let $U = I - \gamma A ^{*}(I - P_{Q})A$, $0<\gamma< 2/\rho(A^{*}A)$, we have

3.25 $$\begin{aligned} \Vert x_{n_{j}} - P_{C}U x_{n_{j}} \Vert & \leq \Vert x_{n_{j}} -P_{C}U_{n_{j}}x_{n_{j}} \Vert + \Vert P_{C}U_{n_{j}}x_{n_{j}} - P_{C}U x_{n_{j}} \Vert \\ & \leq \Vert x_{n_{j}} - P_{C}U_{n_{j}}x_{n_{j}} \Vert + \Vert U_{n_{j}}x_{n_{j}} - Ux_{n_{j}} \Vert \\ & \leq \Vert x_{n_{j}} - P_{C}U_{n_{j}}x_{n_{j}} \Vert + \vert \gamma_{n_{j}}-\gamma \vert M \\ & \rightarrow0, \quad \mbox{as } j\rightarrow\infty. \end{aligned}$$

Since $P_{C}U$ is nonexpansive, from Lemma 2.2, we know that $\overline{x}\in \operatorname{Fix}(P_{C}U)$, that is, $\overline{x}\in\Omega $. It follows from the properties of the projection operator that

3.26 $$ \limsup_{n\rightarrow\infty}\langle x_{n} - q, u-q \rangle= \langle \overline{x}-q, u-q\rangle\leq0. $$

*Step 4.* Finally, we prove $x_{n} \rightarrow q$, where $q = P_{\Omega}u$. By (3.17) and Proposition 2.1, we have

3.27 $$\begin{aligned} \Vert x_{n+1} -q \Vert ^{2} =& \bigl\Vert (1-\alpha_{n})x_{n} + \alpha_{n} \bigl(t_{n} u+(1-t_{n})P_{C}U_{n} x_{n}\bigr) - q \bigr\Vert ^{2} \\ = &\bigl\Vert (1-\alpha_{n}) (x_{n} -q) + \alpha_{n} \bigl(t_{n} (u-q) + (1-t_{n}) (P_{C}U_{n}x_{n} -q) \bigr) \bigr\Vert ^{2} \\ \leq&\bigl\Vert (1-\alpha_{n}) (x_{n} -q)+ \alpha_{n} (1-t_{n}) (P_{C}U_{n} x_{n} -q)\bigr\Vert ^{2} \\ &{} + 2 \alpha_{n} t_{n} \langle u-q, x_{n+1}-q \rangle \\ \leq&(1-\alpha_{n})\Vert x_{n} -q\Vert ^{2} + \alpha_{n} \bigl\Vert (1-t_{n}) (P_{C}U_{n} x_{n}-q)\bigr\Vert ^{2} \\ &{} + 2 \alpha_{n} t_{n} \langle u-q, x_{n+1}-q \rangle \\ \leq&(1-\alpha_{n})\Vert x_{n} -q\Vert ^{2} + \alpha_{n} (1-t_{n})^{2} \Vert x_{n}-q\Vert ^{2} \\ &{} + 2 \alpha_{n} t_{n} \langle u -q,x_{n+1}-q \rangle \\ \leq&(1-\alpha_{n} t_{n})\Vert x_{n} -q \Vert ^{2} + 2 \alpha _{n} t_{n} \langle u -q, x_{n+1}-q\rangle. \end{aligned}$$

It is clear that all conditions of Lemma 2.4 are satisfied. Therefore, we immediately obtain $\Vert x_{n} -q\Vert \rightarrow 0$ as $n\rightarrow\infty$, *i.e.*, $\{x_{n}\}$ converges strongly to $q=P_{\Omega}u$. This completes the proof. □

### Remark 3.1

The results of Dang and Gao [23] is a special case of Theorem 1 by letting $u=0$ in (1.12). Theorem 1 and Theorem 2 consider the variable step sizes of $\{\gamma_{n}\}$, which improve the results of Xu [17], Wang and Xu [22], Dang and Gao [23] and Yu *et al.* [37] where the iterative sequence (1.5), (1.8), and (1.9) are involved with a constant step size of *γ*. Theorem 1 and Theorem 2 also improve the corresponding results of Wang and Xu [24] by discarding the condition of (iv) in (1.10) and (1.11) and weakening the condition on $\{\gamma_{n}\}$ from $\sum_{n=0}^{\infty }\vert \lambda_{n+1}-\lambda_{n}\vert < +\infty$ to $\lim_{n\rightarrow\infty } \vert \gamma_{n+1}-\gamma_{n}\vert =0$.

## Conclusions

The split feasibility problem has been received much attention in recent years. We developed several new iterative algorithms to solve the split feasibility problem in an infinite-dimensional Hilbert spaces. We proved that the iterative sequence converged to the solution of the split feasibility problem which is the best close to a given point. The minimum solution of it can also be found by letting the given point to be zero. Our results improve and generalize the corresponding results of Xu [17], Wang and Xu [22], Dang and Gao [23] and Yu et al. [37].
